# Lactation Ketoacidosis: A Systematic Review of Case Reports

**DOI:** 10.3390/medicina56060299

**Published:** 2020-06-17

**Authors:** Abdullah M. Al Alawi, Asma Al Flaiti, Henrik Falhammar

**Affiliations:** 1 Department of Medicine, Sultan Qaboos University Hospital, Muscat 123, Oman; 2 Oman Medical Specialty Board, Muscat 123, Oman; asma.alflaiti@gmail.com; 3 Division of Medicine, Royal Darwin Hospital, Darwin NT 0800, Australia; henrik.falhammar@ki.se; 4 Department of Endocrinology, Metabolism and Diabetes, Karolinska University Hospital, 171 77 Stockholm, Sweden; 5 Department of Molecular Medicine and Surgery, Karolinska Institute, 171 77 Stockholm, Sweden; 6 Menzies School of Health Research, Darwin NT 0800, Australia

**Keywords:** lactation ketoacidosis, bovine ketoacidosis, lactation ketonemia, breastfeeding, high anion gap metabolic acidosis, ketosis, starvation

## Abstract

*Background and Objective*: Lactation ketoacidosis is a rare cause of high anion gap metabolic acidosis affecting breastfeeding mothers. We aim to review and analyze all cases of lactation ketoacidosis reported. *Materials and Methods*: A systematic search of PubMed/MEDLINE and Cumulative Index to Nursing and Allied Health Literature (CINAHL), identifying relevant case reports published from 1 January 1970 to 31 December 2019. We extracted the following data: the first author, country, year of publication, age of the mother, age of the child, weight/body mass index (BMI) of the mother, precipitating factors, presenting symptoms, biochemical results, treatment, breastfeeding, and time from presentation to the resolution of ketoacidosis. *Results*: Sixteen case reports and 1 case series reporting 18 cases of lactation ketoacidosis were found. Presenting symptoms were nausea (72%, 13/18), vomiting (67%, 12/18), malaise (56%, 10/18), abdominal pain (44%, 8/18), dyspnea (33%, 6/18), headache (22%, 4/18), and palpitation (11%, 2/18). Dieting and physical exercise to lose weight were reported in 76% (14/18). The treatments included IV dextrose, sodium bicarbonate, insulin, rehydration, monitoring and replacement of electrolytes, and resumption of a balanced diet. The prognoses were good, with no mortalities. *Conclusions:* lactation ketoacidosis should be suspected in unwell breastfeeding women with high anion gap metabolic acidosis, after excluding other causes.

## 1. Introduction

Metabolic acidosis is a common medical problem, especially in critically ill patients, and is characterized by low blood pH and serum bicarbonate concentration [1,2]. Metabolic acidosis can occur as a result of the accumulation of acid (high anion gap metabolic acidosis) or loss of bicarbonate from the kidneys or gastrointestinal tract [1,3]. Common causes of high anion metabolic acidosis include diabetic ketoacidosis, lactate, renal failure, and toxins (e.g., methanol, ethanol, and salicylate) [1]. Diarrhea, urinary tract diversions to the intestine, some types of renal tubular acidosis, and some medications are among the common causes of hyperchloremic or normal anion gap metabolic acidosis [1,2].

Insulin inhibits ketogenesis, while epinephrine and glucagon enhance the mobilization of free fatty acid and production of ketone bodies, which can cause high hydrogen load and high anion gap metabolic acidosis, commonly seen in diabetic ketoacidosis but less frequently in starvation and alcoholic ketoacidosis [1,2,3]. Lactation ketoacidosis is a rare cause of high anion gap metabolic acidosis reported in lactating women; however, it is well described in veterinary medicine [4,5]. It may present with non-specific symptoms; however, it is essential to diagnose lactation ketoacidosis and to initiate the appropriate treatment to avoid potentially serious complications, such as cardiac arrhythmia and death [1,5].

In 1983, Chernow et al. first reported the case of lactation ketoacidosis in humans [4]. The case was a 19-year-old lactating woman admitted to hospital with nausea, vomiting, and abdominal pain. Her laboratory workup showed high anion gap metabolic acidosis with ketonuria. The patient was on a low-calorie diet and she had lost 12 kg over the preceding 5 weeks. The ketoacidosis was resolved within 24 hours of the initiation of treatment (IV saline, 5% dextrose, and insulin, in addition to a balanced diet). Due to the lack of any summary of the condition, we undertook this systematic review with the aim to clarify the characteristics and outcomes of lactation ketoacidosis.

## 2. Material and Methods

### 2.1. Search Strategy

A systematic search of PubMed/MEDLINE and Cumulative Index to Nursing and Allied Health Literature (CINAHL) was conducted to identify relevant reports, in all languages, published from 1 January 1970 to 31 December 2019. The following terms were searched in isolation and in combinations: “lactation ketoacidosis”, “bovine ketoacidosis”, “lactation ketonemia”, “breastfeeding”, “ketosis”, and “high anion gap metabolic acidosis” (Table 1).

The search was performed independently by two reviewers (A.A. and H.F.). Reports were initially screened for relevance by the titles, and then by the abstracts. Then, potentially relevant reports were included for full-text review. Additionally, a manual search of the reference lists of the relevant articles was performed to identify additional reports.

### 2.2. Study Selection and Data Extraction

Studies including cases with lactation ketoacidosis in humans were included. The following information was extracted when available from each report: the first author, country, year of publication, age of the mother, age of the child, weight of the mother, precipitating factors, presenting symptoms, treatment, management of breastfeeding, and time from presentation to the resolution of acidosis. Additionally, the results of the following investigations were extracted: blood pH and bicarbonate, plasma glucose level, and urine or serum ketones.

We used the tool suggested by Murad et al. to assess the methodological quality and synthesis of the case reports and case series [6]. In total, there were 8 questions—one point for each question—to assess the selection, ascertainment, causality, and reporting of the case reports and case series. We omitted questions 5 and 6 because they were relevant only for cases reporting adverse drug events. In summary, the possible maximum score was 6 for a good quality case. Additionally, we followed the PRISMA (preferred reporting items for systematic reviews and meta-analyses) guidelines for conducting and reporting this review [7].

### 2.3. Statistical Analysis

All values for the biochemical variables were converted to International System of Units (SI) units. Categorical variables were reported as numbers and percentages. Continuous variables were expressed as mean for normally distributed data or median for non-normally distributed data.

## 3. Results

**Figure 1:**
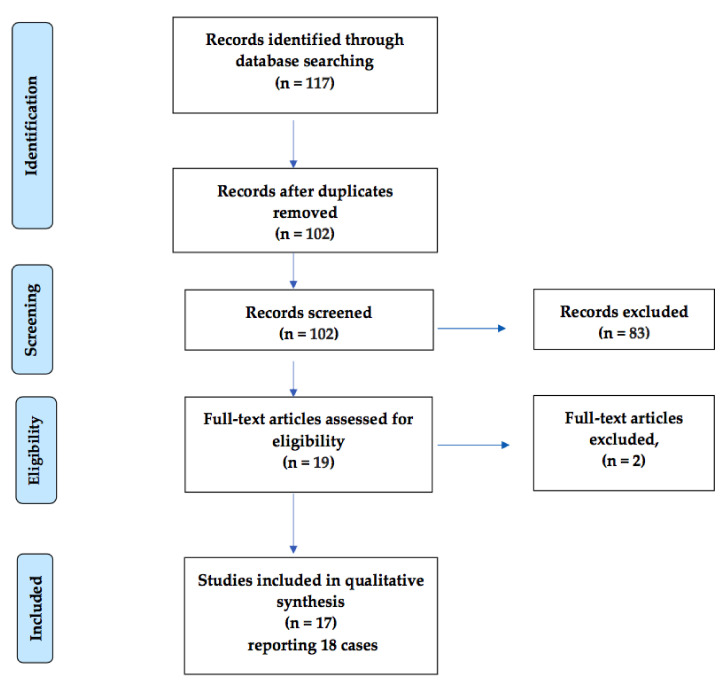
Flowchart illustrating the procedure for article inclusion and exclusion in a systematic review of lactation ketoacidosis.

The systematic searches identified 117 potentially relevant records. After removing 15 duplicated articles, 102 articles were included for screening. Of these, 83 irrelevant articles were excluded after screening the titles and abstracts, and another two articles were excluded after assessing the full articles (Figure 1).

In the end, 17 articles, 16 case reports, and 1 case series reporting on 18 patients with lactation ketoacidosis were included (Table 2 and Table 3) [4,5,8,9,10,11,12,13,14,15,16,17,18,19,20,21,22]. All of these case reports and case series met the criteria for good quality.

### 3.1. Characteristics and Clinical Presentation of the Mothers

The median age of the mothers was 31 years, while the median age of children was 12 weeks. The weight or body mass index (BMI) of the mothers was reported in 9/18 cases, and they seemed normal (BMI: 24.9 kg/m^2^, weight: 62 kg). Two cases (11%) reported mothers who were breastfeeding two babies simultaneously (Table 3). The presenting symptoms were nausea (72%, 13/18), vomiting (67%, 12/18), malaise (56%, 10/18), abdominal pain (44%, 8/18), dyspnea (33%, 6/18), headache (22%, 4/18), and palpitation (11%, 2/18) (Table 2).

### 3.2. Biochemical Characteristics of the Mothers

All patients had high anion gap metabolic acidosis (pH 7.11, HCO3 5.9), with detectable ketones in the blood or urine. About 56% (10/18) of the mothers had hypoglycemia (plasma glucose < 4.0 mmol/L) at presentation, and the median plasma glucose level was 3.8 mmol/L (Table 3).

### 3.3. Precipitating Factors

In almost all cases, there were factors described to precipitate ketoacidosis (Table 2). Recent changes in diet and dieting were described in 76% of cases. A low-calorie diet, low carbohydrate diet, high protein diet, or ketogenic diet were the most common reported diets in the majority of the cases. Intentional weight loss in the weeks leading to the lactation ketoacidosis was reported in some cases [4,5,14,21,22]. Breastfeeding of two babies simultaneously was thought to be a contributing factor for lactation ketoacidosis in two cases [10,21]. Other precipitating factors were gastroenteritis [18,19], severe gastroesophageal reflux, prolonged preoperative fasting [12], significant weight loss after gastric banding surgery [14], and urinary tract infection [4].

### 3.4. Treatment

Intravenous (IV) dextrose was the primary treatment in 89% (16/18) of the patients (Table 2). IV sodium bicarbonate was administered in 44% (8/18) of the patients (Table 1) [9,11,12,13,14,18,19,20]. The patients with severe acidosis, i.e., lower pH, were more likely to be given IV sodium bicarbonate. However, there was no significant difference in the time needed for the resolution of the ketoacidosis between both groups. IV insulin was administered to three patients to suppress ketogenesis [4,16,20], but it was not associated with early resolution of ketoacidosis. In addition, other treatments included the rehydration, monitoring, and replacement of electrolytes, treating the underlying cause, and resumption of a regular diet.

### 3.5. Breastfeeding

Information regarding the breastfeeding status of the mothers after the diagnosis was described in 15 cases, in which 9 mothers (60%) temporarily discontinued breastfeeding and 6 mothers (40%) continued breastfeeding (Table 2). There was no difference in the time needed for the resolution of ketoacidosis between both groups.

### 3.6. Prognosis

The median time required for the resolution of the ketoacidosis from the initiation of treatment was 24 hours (Table 2). Only one patient had a reoccurrence of the condition, but that happened because the diagnosis was missed at the initial presentation [8]. There were no mortalities reported.

## 4. Discussion

Metabolism includes several biochemical processes facilitated by several hormones and enzymes to obtain energy and synthesize functional and structural components required for survival [23]. Metabolism has two main pathways: anabolism, which includes synthesis of macromolecules—such as protein, glycogen, and lipid—and catabolism, which involves the breakdown of these macromolecules into their basic precursors—such as glucose, amino acids, glycerol, and fatty acids. There are several hormones involved in anabolism, including insulin, growth hormone, ghrelin, leptin, and androgens [24,25,26]. In contrast, glucagon, epinephrine, adrenocorticotropic hormone (ACTH), and cortisol are the major hormones during catabolism. The rise in blood glucose level after meals enhances insulin secretion while suppressing glucagon secretion, which facilitates glycogenesis in the liver and muscles [24,27,28]. Several hours after a meal, when blood glucose level drops, glucagon, epinephrine, and other catecholamines increase glucose production through the breakdown of stored glycogen (glycogenolysis /gluconeogenesis) and fatty acids through hydrolysis of triacylglycerols (via hormone-sensitive lipase) [23,24,29]. If fasting continues, the low level of insulin and high level of counterregulatory hormones (glucagon, epinephrine, and other catecholamines) enhance the activity of hormone-sensitive lipase, mobilization and β-oxidation of fatty acids, and production of ketone bodies to a level around 1 mmol/L, which becomes the primary source of energy for the central nervous system [30,31].

During a period of prolonged fasting, ketogenesis continues to produce ketone bodies, peaking at around 20 days of continuous fasting at 8 to 10 mmol/L, which results in a fall in bicarbonate concentration by 7 to 8 mEq/L and rise in anion gap to a similar degree [32,33]. In normal fasting circumstances, the rate of the production of ketone bodies by the liver matches the rate of the utilization of ketone bodies required by the brain and other organs to prevent significant metabolic acidosis [31]. However, fasting beyond 3 weeks (starvation) or the presence of an additional stressor, such as pregnancy, infection, or trauma, accelerates the process of ketogenesis, which can cause significant metabolic acidosis, i.e., a bicarbonate level < 18 mmol/L and anion gap > 18 mmol/L [34,35].

In cows, during late pregnancy, glucose is directed to the nutrition of the calf, which deprives cows of carbohydrates storage [36,37]. At least 50 grams of glucose are required to make 1 liter of cow milk, a demand for glucose which increases when lactation commences [37]. A combination of increased glucose demands and depleted carbohydrate stores accelerates gluconeogenesis and causes intense fatty acid mobilization and ketosis [38,39].

In humans, it is estimated that the lactating woman requires an additional 400–500 kcal per day to support milk production during the first 6 months after delivery [40,41]. Negative energy balance, due to any cause, accelerates ketogenesis in breastfeeding women and may result in lactation ketoacidosis [5].

Our analysis showed that common symptoms of lactation ketoacidosis were nausea, vomiting, abdominal pain, malaise, dyspnea, and headache. These symptoms could be a result of metabolic acidosis and hypoglycemia, but they are non-specific symptoms which can be seen in many medical conditions. The negative energy balances were a result of reduced food intake or physical or physiological stress, while ongoing breastfeeding facilitated fatty acid oxidation and ketogenesis [42]. When the rate of ketogenesis exceeds the buffer capacity of the kidneys, metabolic acidosis occurs [28,43].

We found that hypoglycemia was common in the analyses of the reported cases of lactation ketoacidosis. In general, the body has protective mechanisms to prevent hypoglycemia during a period of energy shortage that includes lowering serum insulin levels and stimulating glucagon, cortisol, epinephrine, and growth hormone secretion [44,45]. These mechanisms result in reduced peripheral use of the glucose, increased hepatic output of the glucose, and synthesis of alternative fuels (i.e., ketone bodies). However, if these mechanisms fail due to an excessive increase in energy demand or severe intercurrent illness, plasma glucose levels continue to fall, and hypoglycemia occurs [44].

Lactation ketoacidosis should be diagnosed after excluding all other causes of high anion gap metabolic acidosis [5] to avoid missing a potentially life-threatening diagnosis that requires specific treatment, such as ethylene glycol and aspirin toxicities [46,47]. For diagnosis of lactation ketoacidosis, a detailed patient history is required, which should cover the pattern of breastfeeding, dietary intake, intensity of physical activities, alcohol, and drug use. Biochemical investigations should include a blood gas to assess for the type and severity of the metabolic acidosis, urine, or serum ketone level, plasma glucose level, bone profile, electrolytes, and kidney function test. Additionally, serum osmolality, drug, and alcohol screening should be considered when there is suspicion of abuse. Screening for infection should be carried out if the history or clinical examination is suggestive of an infective process being the precipitating factor [19]. Based on our experience and the current systematic review, we suggest that certain criteria, listed in Table 4, should be fulfilled before diagnosing lactation ketoacidosis.

Lactation ketoacidosis is more common in lactating animals compared to humans, and some animal studies compare different treatment approaches [37,38]. However, given the rarity of lactation ketoacidosis in humans, there is no randomized trial or even guideline for its management. Our study found that intravenous dextrose was given in the majority of the cases. Dextrose treats the hypoglycemia, provides energy substrate required for metabolism, and stimulates insulin secretion while suppressing glucagon secretion. A high insulin/glucagon ratio suppresses ketogenesis and fatty acid breakdown [24]. Intravenous dextrose is oxidized to yield water and carbon dioxide and supply the body with 3.4 cal/g of d-glucose. There were different strengths of IV dextrose used in the previously reported cases. In general, the high strength of dextrose (20–50%) should be used to treat hypoglycemia, while 5–10% dextrose should be used for hydration and as a maintenance fluid therapy. Additionally, sodium bicarbonate was used in some cases [9,11,12,13,18,19,20]. Administration of sodium bicarbonate was not associated with early resolution of ketoacidosis. However, metabolic acidosis, in general, can cause depressed cardiac function, arrhythmia, hypotension, and altered oxygen delivery [2]. Based on previous studies for the management of acute metabolic acidosis, we suggest considering IV sodium bicarbonate only for the treatment of severe lactation ketoacidosis (i.e., pH < 7.1) [48,49,50]. Moreover, insulin was used in a few cases of lactation ketoacidosis [4,16,20]. Insulin suppresses ketogenesis and fatty acid breakdown [25]. However, the administration of insulin requires close monitoring, and it might be associated with an increased risk of hypoglycemia, especially with the depleted glycogen status.

Electrolyte derangements were common among patients with lactation ketoacidosis [21] and should be monitored and replaced until the patient recovers from metabolic acidosis and is able to resume a balanced diet.

The majority of the patients had lactation ketoacidosis due to a new diet or altered dietary, so resuming a balanced diet and a dietician review are essential. In addition, it is vital to look for other precipitating factors, such as infection [4,18], gastroesophageal reflux, and intestinal obstruction, and treat them as needed. Discontinuation of breastfeeding helps in restoring positive energy balance, but there was no difference in the time required for the resolution of ketoacidosis between the two groups. The presence of milk formula intolerance can complicate the decision to discontinue breastfeeding [5,22]. The decision to discontinue breastfeeding should take into account the severity of the maternal illness, anticipated recovery period, and availability of alternative feeding options for the baby. We would recommend a temporary discontinuation of breastfeeding in women with severe metabolic acidosis (i.e., pH < 7.1), and in the presence of severe illness with an anticipated prolonged period of recovery.

In summary, lactation ketoacidosis is probably an underreported and underdiagnosed cause of high anion gap metabolic acidosis. It is the result of negative energy balance, mainly because of dieting or exercise, in breastfeeding women who may present with non-specific symptoms. Other causes of high anion gap metabolic acidosis should be excluded. Dextrose, hydration, replacement of electrolytes, commencement of a balanced diet, and treatment of the underlying cause are the main treatments. In selected cases with severe metabolic acidosis, sodium bicarbonate could be considered.

This was a systematic review of a limited number of case reports; therefore, reporting and publication bias may profoundly influence the results, including the statistical analysis. The precipitating factors were presumed by the authors of each case report/series and may have been identified incorrectly. Conducting a prospective observational study or retrospective chart review would be a better way to characterize this rare disease.

## 5. Conclusions

The majority of cases recovered within 24 h of the initiation of treatment. The prognoses were excellent, and reoccurrence is unlikely with a balanced energy intake and expenditure.

## Figures and Tables

**Table 1:** Strategy (PubMed).

lactation ketoacidosis bovine ketoacidosis lactation ketonemia 1 OR 2 OR 3 breastfeeding AND ketosis breastfeeding AND high anion gap metabolic acidosis

**Table 2:** Clinical presentation and management approaches of women with lactation ketoacidosis.

#	Author, Year	Presenting Symptoms	Specific Treatment	Precipitating Factors	Breast Feeding After Diagnosis
1	Chemow 1982 [4]	Nausea, vomiting, abdominal pain, and dysuria	IV 0.9% Saline IV 5% Dextrose Insulin	Weight reduction diet (had lost 12 kg) and urinary tract infection	NR
2	Altus 1983 [9]	Vomiting, nausea, rapid breathing, and dehydration	IV 0.9% and 0.45% Saline Dextrose Sodium bicarbonate	High protein, low carbohydrate diet	NR
3	Heffner 2008 [10]	Fatigue and dyspnea, palpitation, and lightheadedness	IV 5% Dextrose IV 0.9% Saline	Altered diet and twin breast-feeding	Continued
4	Sandhu 2009 [11]	Nausea and vomiting, malaise, and dyspnea	IV Dextrose IV Sodium bicarbonate. IV 0.9% Saline	High protein, carbohydrate-free meals	Discontinued
5	Szulewski 2012 [12]	Nausea, vomiting, and abdominal pain	IV Dextrose IV Sodium bicarbonate.	Preoperative prolonged fasting	Discontinued
6	Von Geijer 2015 [16]	Nausea, vomiting, heart palpitation, and tremble	IV 10% Dextrose Insulin	Low carbohydrate, high fat diet	NR
7	Wuopio 2015 [15]	Nausea, dyspnea, and headache	IV Ringer lactate IV Dextrose	Low carbohydrate diet	Discontinued
8	Hudak 2015 [13]	Nausea and vomiting	IV Sodium bicarbonate IV 20% Dextrose IV 0.9% Saline	Reduced oral intake, nausea, and vomiting	Discontinued
9	Monnier 2015 [14]	Dyspnea, fatigue, weight loss, and anorexia	IV Sodium bicarbonate IV Dextrose IV 0.9% Saline	Adjustable gastric band surgery with significant weight loss and gastritis	Discontinued
10	Greaney 2016 [17]	Malaise and vomiting	IV 0.9% Saline IV Dextrose	High protein, low carbohydrate diet and skipped meals	Discontinued
11	Gleason 2016 [8]	Nausea, fatigue, vertigo, malaise, and vomited once	IV 0.9% Saline	Not identified	Discontinued
12	Sloan 2017 [18]	Nausea and vomiting	IV 5% Dextrose IV 0.9% Saline IV Sodium bicarbonate	Low carbohydrate diet and gastroenteritis	Discontinued
13	Al Alawi 2018 [5]	Malaise and headache	IV Dextrose	Altered diet and skipping meals and exercise	Continued
14	Nnodum 2019 [20]	Nausea, vomiting, abdominal pain, diarrhea, and malaise	IV 0.9% Saline 5% Dextrose IV Sodium bicarbinate IV insulin	Low carbohydrate diet	Continued
15	Azzam 2018 [19]	Dyspnoea, headache, fever, vertigo, and vomiting	IV 0.9% Saline 5% dextrose IV Sodium bicarbonate	Gastroenteritis	Continued
16	Seaton 2019 [21]	Nausea, vomiting, and abdominal cramping	Oral feeding and hydration	Ketogenic diet and weight loss (11 kg)	Discontinued
17	Al Alawi 2019 [22]	Lethargy, crampy abdominal pain, and nausea	IV 0.9% Saline IV Dextrose	Altered diet: high protein, glucose- and gluten-free diet	Continued
18	Al Alawi 2019 [22]	Headache, severe malaise, and epigastric pain	IV 0.9% Saline IV Dextrose	Severe GERD and skipping lunch meal	Continued

NR, not reported. IV, intravenous. GERD, gastroesophageal reflux disorder.

**Table 3:** Clinical and biochemical characteristics of women with lactation ketoacidosis.

#	Author, year	Country	Weight (kg) or BMI (kg/m^2^)	Age of Mother (years)	Age of Infant (weeks)	pH	Bicarbonate (mmol/L)	Glucose (mmol/L)	Ketones	Time to Normalization of Acid–Base Balance (hours)
1	Chemow 1982 [4]	USA	NR	19	7	7.25	10	11	Urine +	24
2	Altus 1983 [9]	USA	NR	30	14	7.07	<5	4.2	Urine +	24
3	Heffner 2008 [10]	USA	NR	35	12 (Twins)	7.24	10	3.8	Serum +	24
4	Sandhu 2009 [11]	USA	59.8 kg	36	5	6.9	<5	7.4	Urine +	24
5	Szulewski 2012 [12]	Canada	NR	35	3	7.15	4	6.3	Serum +	48
6	Von Geijer 2015 [16]	Sweden	NR	32	43.5	7.2	NR	3.8	Serum +	24
7	Wuopio 2015 [15]	Sweden	67 kg, BMI 24.8	21	6	6.92	NR	4.4	Serum +	24
8	Hudak 2015 [13]	Germany	NR	32	3	6.99	3	3.8	Urine +	24
9	Monnier 2015 [14]	France	BMI 34	29	26	7.11	2	3.9	Urine +	NR
10	Greaney 2016 [17]	Ireland	85 kg	36	9	6.88	5.8	5.7	Serum +	8
11	Gleason 2016 [8]	New Zealand	NR	31	43.5	7.26	13.5	3.8	Serum +	24
12	Sloan 2017 [18]	UK	BMI 23	27	8	7.02	5.1	3.6	Serum	34
13	Al Alawi 2018 [5]	Australia	61 kg	35	21.7	7.13	9.4	2.9	Serum +	48
14	Nnodum 2019 [20]	USA	BMI 25	22	4.30	7.07	7	NR	Serum +	24
15	Azzam 2018 [19]	Australia	NR	31	12	7.05	5	4.3	Serum + Urine +	15
16	Seaton 2019 [21]	USA	NR	24	18	7.11	6	3.8	Serum +	24
17	Al Alawi 2019 [22]	Australia	57.2 Kg	35	20	7.38	12.3	2.9	Serum +	24
18	Al Alawi 2019 [22]	Oman	63 kg	30	52	7.21	14.9	2.9	Urine +	24

NR: not reported; BMI: body mass index.

**Table 4:** Criteria for diagnosis of lactation ketoacidosis proposed by the authors.

	Presence of High Anion Gap Metabolic Acidosis
and	Positive Urine or Serum Ketones.
and	Current Breastfeeding Status.
and	Excluding Other Causes of High Anion Gap Metabolic Acidosis.
